# Bandwidth Tunable Optical Bandpass Filter Based on Parity-Time Symmetry

**DOI:** 10.3390/mi13010089

**Published:** 2022-01-07

**Authors:** Bowen Zhang, Nuo Chen, Xinda Lu, Yuhang Hu, Zihao Yang, Xinliang Zhang, Jing Xu

**Affiliations:** School of Optical and Electronic Information, Huazhong University of Science and Technology, Wuhan 430074, China; M202072632@hust.edu.cn (B.Z.); nuochen@hust.edu.cn (N.C.); xindalu@hust.edu.cn (X.L.); yuhanghu@hust.edu.cn (Y.H.); m202172642@hust.edu.cn (Z.Y.); xlzhang@mail.hust.edu.cn (X.Z.)

**Keywords:** PT symmetry, integrated optical tunable filter, microresonators

## Abstract

A chip-scale tunable optical filter is indispensable to meeting the demand for reconfigurability in wavelength division multiplexing systems, channel routing, and switching, etc. Here, we propose a new scheme of bandwidth tunable band-pass filters based on a parity-time (PT) symmetric coupled microresonator system. Large bandwidth tunability is realized on the basis of the tuning of the relative resonant frequency between coupled rings and by making use of the concept of the exception point (EP) in the PT symmetric systems. Theoretical investigations show that the bandwidth tuning range depends on the intrinsic loss of the microresonators, as well as on the loss contrast between the two cavities. Our proof-of-concept device confirms the tunability and shows a bandwidth tuning range from 21 GHz to 49 GHz, with an extinction ratio larger than 15 dB. The discrepancy between theory and experiment is due to the non-optimized design of the coupling coefficients, as well as to fabrication errors. Our design based on PT symmetry shows a distinct route towards the realization of tunable band-pass filters, providing new ways to explore non-Hermitian light manipulation in conventional integrated devices.

## 1. Introduction

Driven by the great need for low-cost, scalable, and low-power-consumption integrated optical systems, functional devices based on silicon photonics have been extensively explored in recent years to host system-level integration for various functionalities, including modulators, filters, isolators, and transceivers [1]. Among them, tunable bandwidth optical filters are at the heart of photonic systems since they provide great flexibility. Microring resonators that feature inherent band-pass/band-stop transmission spectra have been widely explored for bandwidth tunable applications. Most designs employ a combination of one cavity, or cascaded cavities, with Mach–Zehnder interferometers (MZI). Chen et al. designed the dual-MZI coupled microrings to achieve a tunable band-pass filter, with a tuning range from 13 GHz to 93 GHz [2], by separately tuning the input and output coupling strengths to control the resonant linewidth and extinction ratio. Ding et al. proposed a structure composed of MZI with coupled rings and realized a ~113-GHz tuning range [3]. Orlandi et al. showed a 150-GHz-bandwidth tuning range with more MZI coupled designs [4]. The system is relatively complicated since it contains multiple thermal-phase tuning structures. An optical filter based on cascaded rings was proposed by Ong et al. [5], reaching a tuning range of 113 GHz using the broadened nature of the synthetic spectra of multiple coupled resonant modes. However, the structure of the multiring cascade brings difficulties to coupling and thermal adjustment, and the in-band ripple is inevitable. Dai et al. employed a four-cascaded microring structure to successfully realize a 225-GHz tuning range filter, with challenges in the severe thermal crosstalk under the large heating power [6]. Poulopoulos et al. proposed a scheme based on two coupled microring resonators, with the coupling regions replaced by Mach–Zehnder interferometers, and a 95-GHz (9–104 GHz) tunable range was achieved. However, six microheaters are required for accessing the bandwidth tuning function [7].

On the other hand, non-Hermitian optics provides a fertile ground for studying unconventional phenomena on integrated platforms [8,9]. Upon the first realization of parity-time (PT) symmetry in an optical system [10], the exotic features of PT symmetry, such as exceptional points (EPs), and spontaneously broken PT symmetry, have been widely investigated to realize increased sensitivity in optical sensors [11], nonreciprocal optical transmission [12], single-mode lasing [13], etc. In this work, we propose a simple design for a tunable bandwidth band-pass filter based on a PT symmetric coupled dual-ring system. Differing from the previous bandwidth tuning mechanism, the linewidth manipulation of the target resonance is realized by adding loss in a non-Hermitian way, resulting in a simplified system design, where only one thermal tuning unit is required. According to theoretical predictions, the bandwidth tuning range depends on the intrinsic loss of the microresonators, as well as on the coupling losses of two cavities, which may become significant if one of the coupling losses is much larger than the intrinsic losses. We note that similar structures are proposed for the design of optical filters [7,14,15], either demonstrated with a tunable central frequency [14,15] or a tunable bandwidth [7]. However, the analyses used in these works are based on the transfer matrix method and the bandwidth tunability is derived by scanning parameters, without a fundamental explanation. In contrast, the tunability of bandwidth in this work is shown using the temporal coupled mode theory. As a result, a clear physical picture behind the coupled microresonators in terms of the PT symmetry is explicitly derived, providing a new guideline for the design of bandwidth-tunable filter. This bandwidth tuning concept is confirmed by theoretical modeling, as well as by experimental measurements of the proof-of-concept devices that have been fabricated on the silicon-on-insulator (SOI) platform.

## 2. Operation Principle

**Figure 1:**
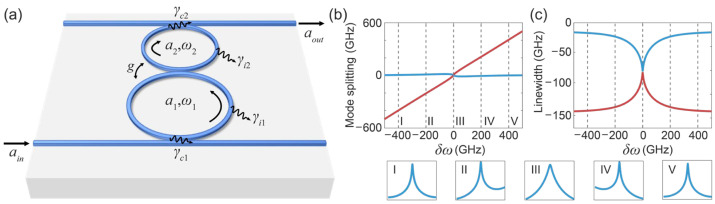
(**a**) Illustration of the PT symmetric coupled dual-ring system; (**b**) Evolution of the mode splitting, i.e., $Re\;\left(\omega_{\pm }\right)-\omega_{1}$, as a function of frequency detuning between two rings, δω; (**c**) Evolution of the resonance linewidths, i.e., $Im\;\left(\omega_{\pm }\right)$, of two rings as a function of δω. I-V are insets of the total system transmission spectra, sampled at different detunings, indicated by the grey dashed lines shown in (**b**,**c**). Parameters used here are: $\gamma_{i1}=\gamma_{i2}=$ 20.7 GHz, $\gamma_{c1}=$ 12.4 GHz, $\gamma_{c2}=$ 398.1 GHz, and g= 93.3 GHz.

The schematic diagram of our design is shown in Figure 1a. Two microrings are coupled to each other, with a coupling rate, g, between the two rings. The intracavity fields and the intrinsic losses of Rings 1 and 2 are denoted as $a_{1}$, $\gamma_{i1}$, and $a_{2}$, $\gamma_{i2}$, respectively. Ring 1 and Ring 2 are coupled to two bus waveguides with field coupling rates, $\gamma_{c1}\;$and $\gamma_{c2}$, respectively. When one of the bus waveguides is used as the input, the other bus waveguide is used as the output. Note that the roles of the waveguides as input or output are interchangeable because of reciprocity [12]. According to the temporal coupled mode theory (TCMT) [12], the evolution of the model field vector, $A={\left(a_{1},a_{2}\right)}^{T}$, is given by $\frac{dA}{dt}=-iHA-\sqrt{\gamma_{c1}}\left(\begin{array}{c} a_{in}\\ 0 \end{array}\right)$, where $a_{in}$ is the input model field, and the non-Hermitian Hamiltonian *H* reads:

(1) $$H=\left[\begin{array}{cc} \omega_{1}-i\frac{\left(\gamma_{i1}+\gamma_{c1}\right)}{2} & g\\ g & \omega_{2}-i\frac{\left(\gamma_{i2}+\gamma_{c2}\right)}{2} \end{array}\right]$$

The eigenfrequencies of *H* are calculated as:

(2) $$\begin{array}{ll} \omega_{\pm }= & \frac{\omega_{1}+\omega_{2}}{2}-i\frac{\left(\gamma_{i1}+\gamma_{i2}+\gamma_{c1}+\gamma_{c2}\right)}{4}\\ & \pm \frac{1}{2}\sqrt{4g^{2}-{\left[-i\left(\omega_{1}-\omega_{2}\right)-\frac{\left(\gamma_{i1}-\gamma_{i2}+\gamma_{c1}-\gamma_{c2}\right)}{2}\right]}^{2}} \end{array}$$

The evolutions of the real part and the imaginary part of the eigenfrequencies, along with a function of the frequency detuning between the two rings, i.e., $\omega_{1}-\omega_{2}$, denoted as δω, are plotted in Figure 1b,c, respectively. The blue lines correspond to the supermode that features less loss, which is the main focus of this work. According to Figure 1c, the imaginary part of eigenfrequencies, i.e., the resonance linewidth of the coupled resonance, changes significantly as the δω varies, achieving a tunable bandwidth operation by varying the δω. By setting $4g\;\approx \;\gamma_{i1}-\gamma_{i2}+\gamma_{c1}-\gamma_{c2}$, the passive PT symmetry system operates near the EP when δω=0. This is important since the mode splitting vanishes at zero detuning while the loss or resonance linewidth is maximized, enabling broadband signal operation without signal distortion. Insets I-V show transmission spectra obtained at five different δω (indicated in Figure 1b,c by the grey dashed lines). A much broader bandwidth achieved in III compared to I and V shows the bandwidth tuning concept of our scheme. According to Equation (2), the maximum bandwidth is $\left(\gamma_{i1}+\;\gamma_{i2}+\gamma_{c1}+\gamma_{c2}\right)/4$ (i.e., $Im\;\left(\omega_{\pm }\right)$ at EP), and the minimum bandwidth is $\left(\gamma_{i1}+\gamma_{c1}\right)/2$. Therefore, the ratio between the maximum and minimum bandwidths depends on the contrast between $\gamma_{c2}$ and $\gamma_{i1}$, assuming that $\gamma_{i1}$, $\gamma_{i2}$ and $\gamma_{c1}$ are comparable and much smaller than $\gamma_{c2}$, which may become significant if $\gamma_{c2}\;\gg \;\gamma_{i1}$.

**Figure 2:**
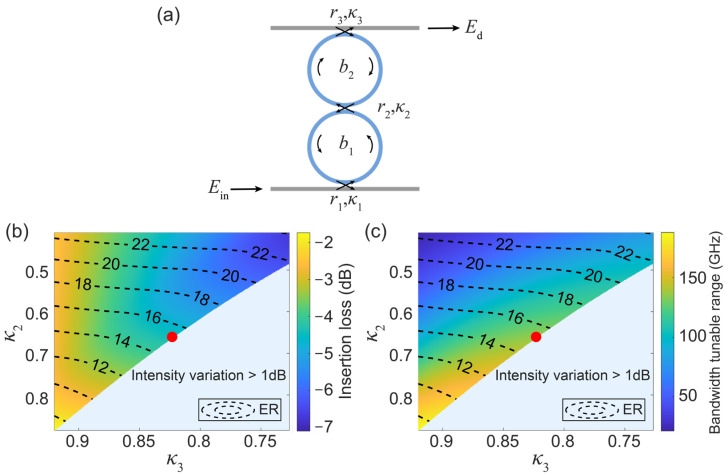
(**a**) Notations used in TMM analysis; (**b**,**c**) Maps of the *IL*/bandwidth tunable range as a function of $\kappa_{2}$ and $\kappa_{3}$. Contour lines represent the *ER*. Red point indicates the best coupling conditions under which the system has low *IL* (−5 dB), high *ER* (15 dB), and a large bandwidth tunable range (about 140 GHz), simultaneously. Parameters used are: $\kappa_{1}=\sqrt{1-{\left[b_{1}\frac{\left(r_{2}+b_{2}r_{3}\right)}{\left(1+r_{2}b_{2}r_{3}\right)}\right]}^{2}}$. $b_{1}$ = 0.9868 and $b_{2}$ = 0.9934.

Although the TCMT provides a clear physical picture that explains the evolution of the bandwidth variation, it is valid in the vicinity of the considered resonance under weak coupling approximation. In order to conduct a complete and rigorous analysis, the transfer matrix method (TMM) [16] is used in the following to investigate the system performances. The notations used in the TMM are illustrated in Figure 2a. That is, $\kappa_{1}$, $\kappa_{2}$, $\kappa_{3}$ are the coupling coefficients between the input waveguide and Ring 1, the two rings, and the upper waveguide and Ring 2, respectively. The corresponding field transmission coefficients, $r_{1}$, $r_{2}$, and $r_{3}$, satisfy the relation, $\kappa_{j}^{2}+r_{j}^{2}=1$, under the lossless approximation, and j=1, 2 and 3. $b_{1}$ and $b_{2}$ are the round-trip transmission coefficients of Rings 1 and 2, respectively. The total system transmission response is calculated as:

(3) $$T={\left|\frac{E_{d}}{E_{in}}\right|}^{2}={\left|-i\kappa_{3}\cdot \frac{-i\kappa_{2}\sqrt{b_{2}}\cdot e^{i\frac{\phi_{2}}{2}}}{1-b_{2}r_{2}r_{3}\cdot e^{i\phi_{2}}}\cdot \frac{-i\kappa_{1}\sqrt{b_{1}}\cdot e^{i\frac{\phi_{1}}{2}}}{1-b_{1}\tau_{1}r_{1}\cdot e^{i\phi_{1}}}\right|}^{2}$$

where $\tau_{1}=\left(r_{2}-b_{2}r_{3}\cdot e^{i\phi_{2}}\right)/\left(1-b_{2}r_{2}r_{3}\cdot e^{i\phi_{2}}\right)$ denotes the field transmission response of the auxiliary ring; $\phi_{i}=2\pi n_{g}L_{i}/\lambda \;\left(i=1,2\right)$ is the round-trip phase accumulation in Ring 1 or 2; $n_{g}$ is the group index of the waveguide; and λ is the vacuum wavelength. The bandwidth tunable range is defined as the difference between the maximum and minimum bandwidths. The extinction ratio (*ER*) is an important parameter of the band-pass filters, defined as $ER=T_{max}/T_{min}$. The insertion loss (*IL*) is $IL=T_{max}$. To find the best system operating point, *IL* and the bandwidth tuning range variations are plotted in Figure 2b,c.

As a function of $\kappa_{2}$ and $\kappa_{3}$ while $\kappa_{1}=\sqrt{1-{\left[b_{1}\frac{\left(r_{2}+b_{2}r_{3}\right)}{\left(1+r_{2}b_{2}r_{3}\right)}\right]}^{2}}$ so that the system operates at the critical coupling point. At the critical coupling point, the coupling coefficient, $\kappa_{1}$ is matched with the system total loss to guarantee that all the injected light is launched into the rings and that it is completely extinct at the straight port of the input waveguide. *ER*s are indicated by the contour lines. The red points are found to be the best coupling conditions in the parameter space, ensuring relatively low *IL* (−5 dB), moderate *ER* (15 dB), and the large bandwidth tunable range (about 140 GHz), simultaneously. Note that this is calculated assuming the quality factor (Q) of the rings is ~2 × 10^5^. The minimum and maximum bandwidths at this point are 28.8 GHz and 166.6 GHz, respectively. The ratio between the maximum and minimum bandwidths can be further increased if the Q factor of the rings can be improved. Note that the light blue areas without contour lines indicate the regime where the intensity variation of the filter response is larger than 1 dB, which has been neglected to ensure a small enough signal distortion.

## 3. Experimental Results

**Figure 3:**
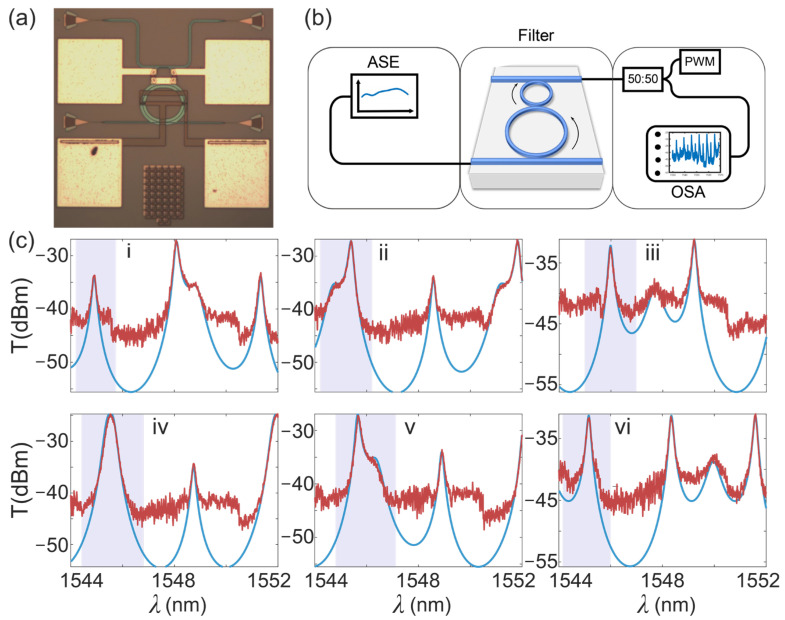
(**a**) Microscope image of the implemented structure; (**b**) Experimental setup. ASE: amplified spontaneous emission light source. PWM: optical power meter. OSA: optical spectrum analyzer; (**c**) Transmission spectra at different applied voltages of the auxiliary ring (**i**–**vi**): voltages applied are 0, 0.8, 1.3, 1.46, 1.66, and 1.86 V, respectively. Red lines are the experimental results, and blue lines are the fitted results based on TMM. Fitting parameters are: $\kappa_{1}=$ 0.5041, $\kappa_{2}=$ 0.2309, $\kappa_{3}=$ 0.6509, $b_{1}=$ 0.9868, and $b_{2}=$ 0.9934. The relations between the applied voltages, the bandwidths, and the wavelength shifts are presented in Table 1.

The designed structure was fabricated using a complementary metal-oxide-semiconductor (COMS) compatible process on an SOI wafer, with a 220 nm-thick top Si layer upon a 2 μm-thick buried dioxide layer. A 3 μm-thick silica layer is deposited on the top by plasma-enhanced chemical vapor deposition (PECVD). Vertical coupling gratings are used as input and output ports. Microheaters are fabricated over the cavities to tune the resonant frequencies of the main ring and the auxiliary ring. A microscope image of the structure is shown in Figure 3a. The perimeters of the main ring and the auxiliary ring are 192.2 μm and 96.4 μm, respectively. Note that the relative size of the auxiliary ring is not critical for the realization of the bandwidth tunability. The gaps between the main ring and the input bus waveguide, the two rings, and the auxiliary ring and the output waveguide are 570 nm, 600 nm, and 390 nm, respectively, corresponding to $\kappa_{1}=\;0.33$, $\kappa_{2}=\;0.28$ and $\kappa_{3}=\;0.71$, accordingly. The conversion between the gap numbers and the coupling coefficients are determined by the coupled mode theory [16], with the optical fields extracted from finite-difference time-domain (FDTD) software (FDTD solutions). Note that the coupling conditions of such a device are not at optimum but can be used to prove the concept of bandwidth tunability. The experimental setup is sketched in Figure 3b. The light from an amplified spontaneous emission (ASE) light source is coupled to the fabricated devices.

In the test using ASE as a light source, no polarization controller (PC) is needed. The injected power of the ASE source is 15.6 mW (11.9 dBm). The output light is further connected to an optical power meter (PWM) and an optical spectrum analyzer (OSA) by a 50:50 optical power splitter. Note that the filter and vertical coupling gratings are designed for TE polarization. The measured extinction ratios between TE mode and TM mode are about 14 dB.

The total insertion loss of our proof-of-principle devices is ~30 dB, which is much larger than our expectation, which is mainly due to the imperfect design of the vertical gratings. Fabrication errors also lead to the consequences of relatively low ER values and system instability. We expected improved performances with optimized design and fabrication. The measured transmission spectra are shown in Figure 3c, where the resonant frequency of the auxiliary ring is tuned by changing the voltages of the microheaters. The corresponding voltage settings are 0, 0.8, 1.3, 1.46, 1.66, and 1.86 V, respectively. As the applied voltage increases, the resonance of the auxiliary ring is scanning across one resonance of the main ring. The relations between the applied voltages, the bandwidths, and the wavelength shifts are presented in Table 1. Consequently, the 3 dB bandwidth of the target resonance, as shown in the shaded regions, changes accordingly. The experimental results agree well with the theoretical predictions (blue lines). The coupling coefficients estimated from the theoretical fittings are $\kappa_{1\;}=$ 0.5041, $\kappa_{2}=$ 0.2309, and $\kappa_{3}=$ 0.6509. Compared with the designed parameters, these coefficients correspond to fabrication errors of about 80 nm, 40 nm, and 30 nm, respectively.

**Figure 4:**
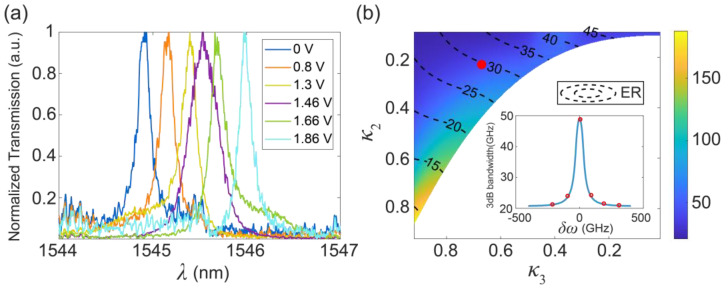
(**a**) Normalized transmission spectra of the fabricated device; (**b**) bandwidth tuning range as a function of $\kappa_{2}$ and $\kappa_{3}$ when $\kappa_{1}=$ 0.5041, which is estimated by fitting the experimental measurements using TMM. The device is operated at the red point. Inset: experimental and fitted 3-dB bandwidths as a function of detuning. Six red circles correspond to (**a**), as well as to Figure 3 (ci–cvi).

To demonstrate the effect of the bandwidth tuning more clearly, Figure 4a shows the normalized transmission spectra on a linear scale. The purple line indicates that two rings are aligned, which has the largest bandwidth compared to the other cases. The measured bandwidth tuning range is from 21 GHz to 49 GHz. The derivation of the bandwidth tuning range from the optimum design is because $\kappa_{1}$ is significantly larger than expected because of fabrication errors. Figure 4b shows the bandwidth tuning range as a function of $\kappa_{2}$ and $\kappa_{3}$ when $\kappa_{1}=$ 0.5041. The fabricated device is operated at the redpoint. The inset of Figure 4b shows the measured (red circles) and simulated (blue lines) bandwidths as a function of detuning, which shows excellent consistency. An *ER* of 30 dB is predicted, according to the theoretical model. The measured *ER* is larger than 15 dB because of the large insertion loss and the existence of the noise floor (Figure 3c). The big insertion loss observed in the experiments is due to the non-optimal design of the vertical grating couplers. The bandwidth tuning range and the *ER* can be optimized by improving the fabrication accuracy and reducing the total insertion loss.

## 4. Conclusions

In conclusion, we theoretically proposed a bandwidth tunable optical band-pass filter by employing a PT symmetric coupled dual-ring system. Loss introduced by the auxiliary ring is used to tune the resonance linewidth. When the two cavities are aligned, the PT symmetric system is operated at EP, resulting in a broad unsplit resonance compared to misaligned cases. Theoretical investigations show that the bandwidth tuning range depends on the intrinsic loss of the microresonators, as well as on the loss contrast between the two cavities. Proof-of-concept devices are fabricated on the SOI platform, achieving a bandwidth tuning range from 21 GHz to 49 GHz via the thermal tuning of the resonant frequency of one of the cavities. The experimental results of the adjustable bandwidth range agree well with the theoretical analysis. The non-optimized results observed in the bandwidth tuning range are due to the non-optimized design, as well as to manufacturing imperfection. Note that our design is simple with respect to thermal tuning, and that it is capable of supporting two channels of light filtering at the same time, i.e., using both waveguides as input and extracting light from the opposite waveguides. Our results show a promising way of utilizing non-Hermitian optics for conventional integrated devices.

## Figures and Tables

**Table 1:** The relations between applied voltages, the bandwidths, and wavelength shifts in Figure 3c.

	i	ii	iii	iv	v	vi
Applied voltage (V)	0	0.8	1.3	1.46	1.66	1.86
Bandwidth (nm)	0.167	0.169	0.194	0.392	0.193	0.169
Wavelength shift (nm)	0	1.19	2.46	3.20	3.94	5.38

## Data Availability

The data presented in this study are available from the corresponding author upon reasonable request.
